# Four New Pale-Spored Species of *Xylaria* (Xylariaceae, Xylariales) with a Key to Worldwide Species on Fallen Fruits and Seeds

**DOI:** 10.3390/biology11060885

**Published:** 2022-06-08

**Authors:** Haixia Ma, Zikun Song, Xiaoyan Pan, Zhi Qu, Zhanen Yang, Yu Li, Anhong Zhu

**Affiliations:** 1 Institute of Tropical Bioscience and Biotechnology, Chinese Academy of Tropical Agricultural Sciences, Haikou 571101, China; michellesong2021@yeah.net (Z.S.); pxy960501@yeah.net (X.P.); quzhi@itbb.org.cn (Z.Q.); fungizhaneny@163.com (Z.Y.); 2 Hainan Institute for Tropical Agricultural Resources, Haikou 571101, China; 3 Hainan Key Laboratory of Tropical Microbe Resources, Haikou 571101, China; 4 College of Plant Protection, Jilin Agricultural University, Changchun 130118, China; liyu@itbb.org.cn; 5 College of Biodiversity Conservation, Southwest Forestry University, Kunming 650224, China; 6 Chinese Academy of Tropical Agricultural Sciences, Haikou 571101, China

**Keywords:** Ascomycota, Xylariaceae, fructicolous fungi, molecular phylogenetics, taxonomy of new species

## Abstract

**Simple Summary:**

*Xylaria*, a large, complex, and cosmopolitan genus of Ascomycota, are known as a source of bioactive secondary metabolites with antibacterial, antioxidative, anti-carcinogenic, and other properties. The species of this genus usually grow on decayed wood, fallen fruits or seeds, fallen leaves or petioles, and termite nests. The present paper describes species of *Xylaria* associated with fruits and seeds using morphological and multigene phylogenetic analyses based on specimens collected in Southwest China. There are few detailed reports on *Xylaria* taxonomy from China, especially on the species associated with fallen fruits and seeds. In this study, we describe four new species from the genus *Xylaria* with pale-colored ascospores on fallen fruits. They are described, illustrated, and compared with morphologically similar species, and their nucleotide sequences of ITS, RPB2, and β-tubulin were obtained and analysed. Our study reports new species of *Xylaria* with pale-colored ascospores associated with fallen fruits and seeds in China for the first time.

**Abstract:**

*Xylaria*, a large and cosmopolitan genus of Ascomycota, plays an important ecological role in forest ecology as wood-decomposers, and serve as a source of bioactive secondary metabolites. The present work concerns a survey of *Xylaria* from Southwest China. Four new species of *Xylaria* with pale-colored ascospores associated with fallen fruits and seeds are described and illustrated based on morphological and phylogenetic evidences. The phylogeny inferred from a combined dataset of ITS-RPB2-β-tubulin sequences supports these four species as distinct species. The four new taxa, namely *Xylaria* *rogersii*, *X. schimicola*, *X. theaceicola*, and *X. wallichii*, are compared and contrasted against morphologically similar species. A dichotomous identification key to all the accepted species of *Xylaria* associated with fallen fruits and seeds is given.

## 1. Introduction

The genus *Xylaria* Hill ex Schrank is one of the most complex and difficult genera in the Xylariaceae. Stromata morphology of many species often vary greatly in color, size, and even in general shape with stages of development. The genus is widely distributed in tropical, subtropical, and temperate regions. More than 300 *Xylaria* species have been reported in the world [1], and more than 800 epithets are listed in Index Fungorum (http://www.indexfungorum.org/, accessed on 1 March 2022) [2]. *Xylaria* species are characterized by having upright, stipitate, woody to leathery stromata with perithecia entirely immersed [3,4].

Most *Xylaria* species inhabit decayed wood, while some grow on fallen fruits or seeds, leaves or petioles, and termite nests. The *Xylaria* species associated with fallen fruits/seeds or leaves/petioles are substrate-specific [5,6,7,8]. Examples include *Xylaria magnoliae* J.D. Rogers on *Magnolia* fruits, *Xylaria xanthinovelutina* Mont. on leguminous pods [6], *X. carpophila* (Pers.) Fr. on *Fagus* fruits, *X. liquidambaris* J.D. Rogers, Y.M. Ju & F. San Martín on *Liquidambar* fruit [9], *X. guareae* Læssøe et Lodge on *Guarea guidonia*, *X. meliacearum* Læssøe on fine litter of trees in the Meliaceae, and *X. axifera* Mont. on fallen petioles of Araliaceae [7]. However, some species are not restricted to fallen fruits or seeds, such as *X. clusiae* K.F. Rodrigues, J.D. Rogers & Samuels, *X. duranii* San Martín & Vanoye, and *X. heloidea* Penz. & Sacc., which can also be found on fallen leaves [5]. Therefore, it is ecologically interesting to study *Xylaria* species occurring on fruits and seeds. Certain *Xylaria* species on fruits and seeds have already been taxonomically studied [5,6,7,9,10,11,12]. Recently, Perera et al. [8] described two new species, *X. fabacearum* R.H. Perera, E.B.G. Jones & K.D. Hyde and *X. fabaceicola* R.H. Perera, E.B.G. Jones & K.D. Hyde, from Thailand, increasing the number of species on fruits and seeds to 29.

In China, about 70 species of *Xylaria* have been reported [13,14,15,16,17,18,19,20,21,22,23], but only four species are associated with fallen fruits and seeds. Teng [13] reported three species of *Xylaria* from fallen fruits and seeds, *X. carpophila*, *X. xanthinovelutina*, and *X. warburgii* Henn., whereas *X. carpophila* was a misidentified specimen. Huang et al. [24] described a new species, *X. beilschmiediae* G. Huang & L. Guo, on fallen fruit of *Beilschmiedia percoriacea* from Southern China. During investigations on the diversity of xylariaceous specimens in Southwest China, four undescribed *Xylaria* species associated with fallen fruits and seeds were collected and taxonomically characterized based on morphological criteria and phylogenetic analyses. The primary purpose of the present study is to use an integrative taxonomic approach for the delimitation and description of four new species of *Xylaria* from China, and to discuss the phylogeny of the genus *Xylaria* based on expanded sampling.

## 2. Materials and Methods

### 2.1. Sample Collection and Morphological Study

Field sampling trips in nature reserves and forest parks in tropical and subtropical regions of Southwest China were carried out by the authors. The photos of the materials were taken with a Canon camera G15 (Canon Corporation, Tokyo, Japan). Fresh specimens were dried with a portable drier (manufactured in China). Dried specimens were labeled and stored in an ultra-low freezer at −80 °C for 1 week to kill insects and their eggs, and then they were ready for morphological and molecular studies. Voucher specimens are deposited in the Fungarium of the Institute of Tropical Bioscience and Biotechnology, Chinese Academy of Tropical Agricultural Sciences (FCATAS). 

Microscopic features and measurements were made from slide preparations mounted in water, Melzer’s iodine reagent, 5% KOH, 1% SDS, and Indian ink. The average range of ascospore size refers to more than 95% of spores, and the extreme values are given in parentheses. In the text, the following abbreviations are used: L = mean ascospore length (arithmetical average of all ascospores); W = mean ascospore width (arithmetical average of all ascospores); M = L × W; Q = L/W ratio; n (a/b) = number of ascospores (a) measured from number of specimens (b). The photographs of asci, ascus apical apparatus, and ascospores were examined by differential interference microscopy (DIC) and bright field microscopy (BF) with a Zeiss Axio Scope A1 (Zeiss Corporation, Oberkochen, Germany) and a scanning electron microscope (SEM) (Hitachi Corporation, Tokyo, Japan), respectively. Stromatal surface and perithecia were observed and photographed using a VHX-600E microscope of the Keyence Corporation (Osaka, Japan). Color codes and names followed Rayner [25].

### 2.2. Molecular Procedures and Phylogenetic Analyses

Total DNA from herbarium specimens was extracted using a cetyltrimethylammonium bromide (CTAB) rapid extraction kit for plant genomes (Aidlab Biotechnologies, Beijing, China) according to the manufacturer’s instructions. Target regions of the ITS rDNA, RPB2, and β-tubulin, were amplified by polymerase chain reaction (PCR) using TaKaRa Taq (TaKaRa Bio, Kusatsu, Japan) and fungal specific primers. Approximately 500 base pairs of the ITS region were amplified with primers ITS5 and ITS4 [26], using the following procedure: initial denaturation at 98 °C for 5 min, followed by 30 cycles of 95 °C for 1 min, 55 °C for 1 min, and 72 °C for 2 min, and a final extension of 72 °C for 10 min. For the RPB2 gene, about 1200 base pairs were amplified with primers fRPB2-5F and fRPB2-7cR [27], using the following procedure: initial denaturation at 95 °C for 5 min, followed by 30 cycles of 95 °C for 1 min, 55 °C for 2 min, and 72 °C for 2 min, and a final extension of 72 °C for 10 min. For the β-tubulin gene, about 1500 base pairs were amplified with primers T1 and T22 [28], using the following procedure: initial denaturation at 95 °C for 2 min, followed by 30 cycles of 95 °C for 1 min, 54–45 °C for 1.5 min, and 72 °C for 2 min, and a final extension of 72 °C for 10 min [29]. DNA sequencing was performed at BGI tech (Guangzhou, China), and all the newly generated sequences were submitted to GenBank (Table 1).

Two separate datasets, the concatenated ITS-RPB2-β-tubulin sequences of *Xylaria* and related genera in the family Xylariaceae, and ITS-only sequences of *Xylaria* from GenBank, were analyzed. *Poronia pileiformis* (Berk.) Fr. was selected as an outgroup [30]. The sequences of ITS, RPB2, and β-tubulin were aligned separately using the MAFFT V.7 online server (https://mafft.cbrc.jp/alignment/server/, accessed on 12 March 2022) [31] with the G-INS-i iterative refinement algorithm, and rechecked and improved manually using BioEdit v. 7.0.5.2 [32]. Phylogenetic analyses were carried out with maximum likelihood (ML) and Bayesian inference (BI) analysis, respectively. The ML analysis was performed using RaxML v.8.2.10 [33] with the bootstrap values obtained from 1000 replicates and the GTRGAMMA model of nucleotide evolution. The BI was performed using MrBayes 3.2.6 [34]. ITS sequences were inferred and used to confirm the *Xylaria* species identification carried out in the study. Phylogenetic trees were viewed in FigTree version 1.4.2 [35].

**Table 1:** List of taxa used for the phylogenetic reconstruction. GenBank accession numbers, specimen numbers, origin, and reference studies are given. Holotype specimens are labeled with HT. Sequences from specimens highlighted in bold are derived from this study. N/A: not available.

Species	Specimen No.	Origin	Host	GenBank Accession Number			Reference
				ITS	RPB2	β-Tubulin	
*Amphirosellinia fushanensis*	HAST 91111209 (HT)	China	dead twigs	GU339496	GQ848339	GQ495950	[36,37]
*A. nigrospora*	HAST 91092308 (HT)	China	dead twigs	GU322457	GQ848340	GQ49595	[36,37]
*Astrocystis mirabilis*	HAST 94070803	China	bamboo culms	GU322448	GQ844835	GQ49594	[36]
*As. sublimbata*	HAST 89032207	China	bamboo culms	GU322447	GQ844834	GQ495940	[36]
*Kretzschmaria guyanensis*	HAST 89062903	China	bark	GU300079	GQ844792	GQ478214	[36]
*K. sandvicensis*	JDR 113	USA	wood	GU300076	GQ844786	GQ478211	[36]
*Nemania abortiva*	BiSH 467 (HT)	USA	decayed wood	GU292816	GQ844768	GQ470219	[36]
*N. diffusa*	HAST 91020401	China	bark	GU292817	GQ844769	GQ470220	[36]
*Podosordaria mexicana*	WSP 176	Mexico	horse dung	GU324762	GQ853039	GQ844840	[36]
*P. muli*	WSP 167 (HT)	Mexico	mule dung	GU324761	GQ853038	GQ844839	[36]
*Poronia pileiformis*	WSP 88113001 (ET)	China	cow dung	GU324760	GQ853037	GQ502720	[36]
*Rosellinia buxi*	JDR 99	France	*Buxus sempervivens*	GU300070	GQ844780	GQ470228	[36]
*R. necatrix*	HAST 89062904	China	root	EF026117	GQ844779	EF025603	[36]
*Xylaria adscendens*	HAST 570	Guadeloupe	wood	GU300101	GQ844817	GQ487708	[36]
*X. aethiopica*	YMJ 1136	Ethiopia	pods of *Millettia ferruginea*	MH790445	MH785222	MH785221	[11]
*X. allantoidea*	HAST 94042903	China	trunk	GU324743	GQ848356	GQ502692	[36]
*X. amphithele*	HAST 529	Guadeloupe	dead leaves	GU300083	GQ844796	GQ478218	[36]
*X. apoda*	HAST 90080804	China	bark	GU322437	GQ844823	GQ495930	[36]
*X. arbuscula*	HAST 89041211	China	bark	GU300090	GQ844805	GQ478226	[36]
*X. atrosphaerica*	HAST 91111214	China	bark	GU322459	GQ848342	GQ495953	[36]
*X. berteri*	HAST 90112623	China	wood	GU324749	GQ848362	AY951763	[36]
*X. betulicola*	FCATAS750 (HT)	China	leaves of *Betula*	MF774332	N/A	N/A	[22]
*X. brunneovinosa*	HAST 720 (HT)	China	ground of bamboo plantation	EU179862	GQ853023	GQ502706	[36,38]
*X. carpophila*	CBS 453.72	Netherlands	-	MH860527	N/A	N/A	[39]
*X. cirrata*	HAST 664 (ET)	China	ground of vegetable farm	EU179863	GQ853024	GQ502707	[36,38]
*X. cranioides*	HAST 226	China	wood	GU300075	GQ844785	GQ478210	[36]
*X. crinalis*	FCATAS751 (HT)	China	wood	MF774330	N/A	N/A	[22]
*X. cubensis*	JDR 860	USA	wood	GU991523	GQ848365	GQ502700	[36]
*X. culleniae*	JDR 189	Thailand	pod	GU322442	GQ844829	GQ495935	[36]
*X. curta*	HAST 92092022	China	bark	GU322443	GQ844830	GQ495936	[36]
*X. digitata*	HAST 919	Ukraine	wood	GU322456	GQ848338	GQ495949	[36]
*X. enterogena*	HAST 785	French Guiana	wood	GU324736	GQ848349	GQ502685	[36]
*X. fabacearum*	MFLU 16-1061 (HT)	Thailand	seed pods of Fabaceae	NR171104	MT212202	MT212220	[8]
*X. fabaceicola*	MFLU 16-1072 (HT)	Thailand	seed pods of Fabaceae	NR171103	MT212201	MT212219	[8]
*X. feejeensis*	HAST 92092013	China	bark	GU322454	GQ848336	GQ495947	[36]
*X. ficicola*	HMJAU 22818	China	leaves and petioles of *Ficus auriculata*	MZ351258	N/A	N/A	[40]
*X. filiformis*	GUM 1052	Iran	herbaceous stem	KP218907	N/A	N/A	[41]
*X. fimbriata*	HAST 491	Martinique	termite nest	GU324753	GQ853022	GQ502705	[36]
*X. fissilis*	HAST 367	Martinique	bark	GU300073	GQ844783	GQ470231	[36]
*X. frustulosa*	HAST 92092010	China	bark	GU322451	GQ844838	GQ495944	[36]
*X.* cf. *glebulosa*	HAST 431	Martinique	fruit	GU322462	GQ848345	GQ495956	[36]
*X. globosa*	HAST 775	Guadeloupe	bark	GU324735	GQ848348	GQ502684	[36]
*X. grammica*	HAST 479	China	wood	GU300097	GQ844813	GQ487704	[36]
*X. griseosepiacea*	HAST 641 (HT)	China	ground of mango orchard	EU179865	GQ853031	GQ502714	[36,38]
*X. guareae*	PR71	Puerto Rico	-	AY909009	N/A	N/A	[42]
*X. haemorrhoidalis*	HAST 89041207	China	bark	GU322464	GQ848347	GQ502683	[36]
*X. hedyosmicola*	FCATAS857	China	leaves of *Hedyosmum orientale*	MZ227023	MZ683407	MZ221183	[40]
*X. hypoxylon*	HAST 95082001	China	wood	GU300095	GQ844811	GQ487703	[36]
*X. intracolorata*	HAST 90080402	China	bark	GU324741	GQ848354	GQ502690	[36]
*X. intraflava*	HAST 725 (HT)	China	ground of bamboo plantation	EU179866	GQ853035	GQ502718	[36]
*X. juruensis*	HAST 92042501	China	*Arenga engleri*	GU322439	GQ844825	GQ495932	[36]
*X. karyophthora*	DRH059	Guyana	seeds of *Chlorocardium* sp.	KY564220	KY564216	N/A	[12]
*X. laevis*	HAST 95072910	China	bark	GU324747	GQ848360	GQ502696	[36]
*X. lindericola*	FCATAS852	China	leaves of *Lindera robusta*	MZ005635	MZ031982	MZ031978	[40]
*X. liquidambaris*	HAST 93090701	China	fruits of *Liquidambar formosana*	GU300094	GQ844810	GQ487702	[36]
*X. meliacearum*	JDR 148	Puerto Rico	petioles and infructescence of *Guarea guidonia*	GU300084	GQ844797	GQ478219	[36]
*X. microceras*	HAST 414	Guadeloupe	wood	GU300086	GQ844799	GQ478221	[36]
*X. montagnei*	HAST 495	Martinique	wood	GU322455	GQ848337	GQ495948	[36]
*X. multiplex*	JDR 259	USA	wood	GU300099	GQ844815	GQ487706	[36]
*X. muscula*	HAST 520	Guadeloupe	dead branch	GU300087	GQ844800	GQ478222	[36]
*X. nigripes*	HAST 653	China	ground of mango orchard	GU324755	GQ853027	GQ502710	[36]
*X. ochraceostroma*	HAST 401 (HT)	China	ground of mango orchard	EU179869	GQ853034	GQ502717	[36,38]
*X. oligotoma*	HAST 784	French Guiana	wood	GU300092	GQ844808	GQ487700	[36]
*X. ophiopoda*	HAST 93082805	China	bark	GU322461	GQ848344	GQ495955	[36]
*X. oxyacanthae*	YMJ 1184	Germany	seeds of *Carpinus betulus*	MF773430	MF773434	MF773438	[5,36]
*X. oxyacanthae*	YMJ 1320	Germany	fruits of *Cornus sanguinea*	MF773431	MF773435	MF773439	[5,36]
*X. palmicola*	PDD 604	New Zealand	fruits of palm	GU322436	GQ844822	GQ495929	[36]
*X. papulis*	HAST 89021903	China	wood	GU300100	GQ844816	GQ487707	[36]
*X. phyllocharis*	HAST 528	Guadeloupe	dead leaves	GU322445	GQ844832	GQ495938	[36]
*X. plebeja*	HAST 91122401	China	trunk of *Machilus zuihoensis*	GU324740	GQ848353	GQ502689	[36]
*X. polymorpha*	JDR 1012	USA	wood	GU322460	GQ848343	GQ495954	[36]
*X. polysporicola*	FCATAS848	China	leaves of *Polyspora hainanensis*	MZ005592	MZ031980	MZ031976	[40]
*X. reevesiae*	HAST 90071609 (HT)	China	fruits of *Reevesia formosana*	GU322435	GQ844821	GQ495928	[36]
*X. regalis*	HAST 920	India	log of *Ficus racemosa*	GU324745	GQ848358	GQ502694	[36]
***X. rogersii***	**FCATAS913**	**China**	**fruits of *Magnolia* sp.**	**MZ648825**	**MZ707119**	**MZ695799**	**This study**
***X. rogersii***	**FCATAS914**	**China**	**fruits of *Magnolia* sp.**	**MZ648826**	**MZ707120**	**N/A**	**This study**
***X. rogersii***	**FCATAS915 (HT)**	**China**	**fruits of *Magnolia* sp.**	**MZ648827**	**MZ707121**	**MZ695800**	**This study**
***X. schimicola***	**FCATAS896 (HT)**	**China**	**fruits of *Schima noronhae***	**MZ648850**	**MZ707114**	**MZ695787**	**This study**
***X. schimicola***	**FCATAS898**	**China**	**fruits of *Schima noronhae***	**MZ648851**	**N/A**	**N/A**	**This study**
*X. schweinitzii*	HAST 92092023	China	bark	GU322463	GQ848346	GQ495957	[36]
*X. scruposa*	HAST 497	Martinique	wood	GU322458	GQ848341	GQ495952	[36]
*X. sicula*	HAST 90071613	China	fallen leaves	GU300081	GQ844794	GQ478216	[36]
*Xylaria* sp. *6*	JDR 258	USA	leaves of *Tibouchina semidecandra*	GU300082	GQ844795	GQ478217	[36]
*X. striata*	HAST 304	China	branch of *Punica granatum*	GU300089	GQ844803	GQ478224	[36]
*X. telfairii*	HAST 90081901	China	bark	GU324738	GQ848351	GQ502687	[36]
***X. theaceicola***	**FCATAS903 (HT)**	**China**	**fruits of *Schima villosa***	**MZ648848**	**MZ707115**	**MZ695788**	**This study**
***X. theaceicola***	**FCATAS904**	**China**	**fruits of *Schima villosa***	**MZ648849**	**N/A**	**N/A**	**This study**
*X. tuberoides*	HAST 475	Martinique	wood	GU300074	GQ844784	GQ478209	[36]
*X. venustula*	HAST 88113002	China	bark	GU300091	GQ844807	GQ487699	[36]
*X. vivantii*	HAST 519 (HT)	Martinique	fruits of *Magnolia* sp.	GU322438	GQ844824	GQ495931	[36]
***X. wallichii***	**FCATAS923**	**China**	**fruits of *Schima wallichii***	**MZ648861**	**MZ707118**	**MZ695793**	**This study**
***X. wallichii***	**FCATAS924**	**China**	**fruits of *Schima wallichii***	**MZ648862**	**N/A**	**MZ695794**	**This study**
***X. wallichii***	**FCATAS911 (HT)**	**China**	**fruits of *Schima wallichii***	**ON222810**	**N/A**	**MZ695797**	**This study**
*X. xanthinovelutina*	HAST 553	Martinique	fruit of *Swietenia macrophylla*	GU322441	GQ844828	GQ495934	[36]

## 3. Results

### 3.1. Molecular Phylogenetic Analysis

**Figure 1:**
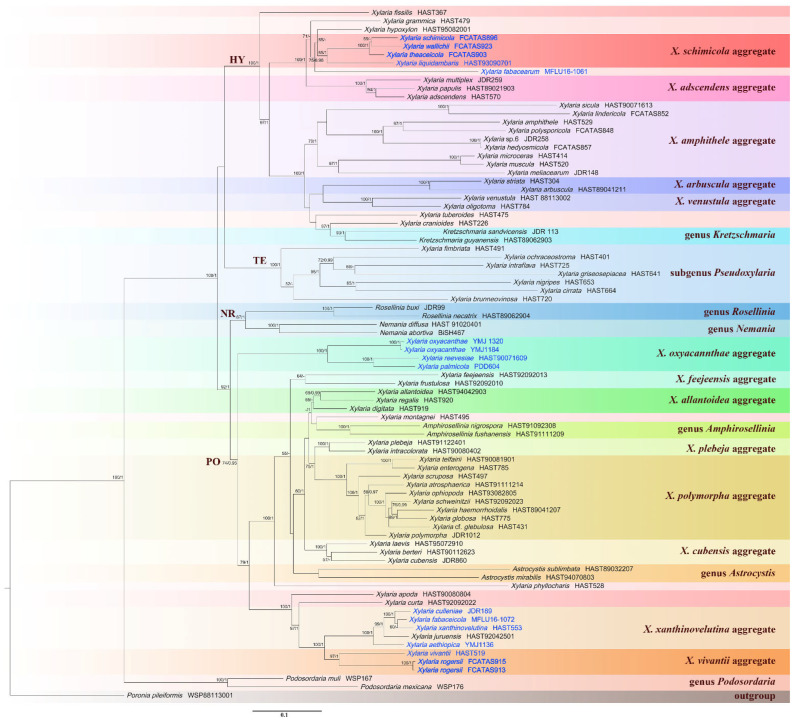
Phylogenetic tree of *Xylaria* and related genera based on the multigene alignment of ITS-RPB2-β-tubulin derived from ML. Support values of ML and BI analyses (bootstrap support ≥50%, posterior probability value ≥0.95) are displayed above or below the respective branches (ML/BI). Species of *Xylaria* associated with fruits and seeds are labeled with blue font.

**Figure 2:**
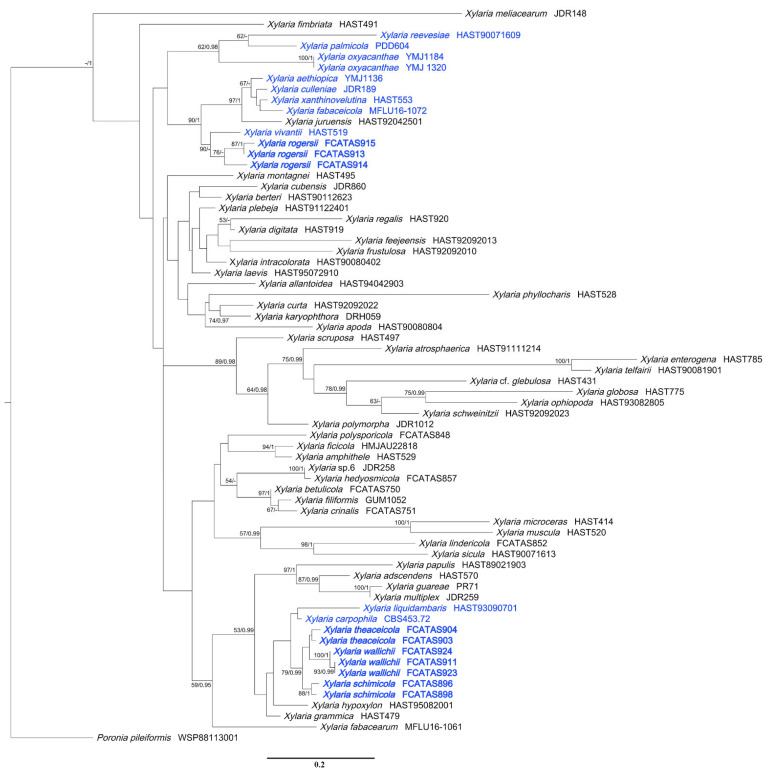
Phylogenetic tree of *Xylaria* based on the dataset of ITS sequences derived from ML. Support values of ML and BI analyses (bootstrap support ≥50%, posterior probability value ≥0.95) are displayed above or below the respective branches (ML/BI). Species of *Xylaria* associated with fruits and seeds are labeled with blue font.

Ten ITS, six RPB2, and seven β-tubulin sequences were generated from this study. The concatenated ITS-RPB2-β-tubulin dataset contained 82 sequences from each gene obtained from 82 samples representing 80 xylariacean taxa and the outgroup (Table 1). The concatenated dataset had an aligned length of 2807 characters, of which 1718 were parsimony-informative. Phylogenetic trees generated from BI and ML analyses of the combined dataset of ITS-RPB2-β-tubulin were highly similar in topology. Only the ML tree is shown in Figure 1 with Bayesian posterior probabilities ≥0.95 and ML bootstrap values ≥ 50% labeled along the branches, while the tree generated by BI analysis is provided in supplementary materials (Figure S1). The ITS dataset contained 68 ITS sequences, representing 62 *Xylaria* taxa with 382 characters, of which 238 were parsimony-informative, and the ML tree is shown in Figure 2.

In the Xylariaceae ITS-RPB2-β-tubulin tree (Figure 1), *Podosordaria* formed a distinct branch separated from *Xylaria* and five other genera, *Amphirosellinia*, *Astrocystis*, *Kretzschmaria*, *Nemania*, and *Rosellinia*. All new *Xylaria* taxa studied in this paper were grouped together with already described species of *Xylaria* associated with fallen fruits and seeds in clades HY and PO. These new species are clearly distinct from each other and from previously known species. *Xylaria* spp. subgenus *Pseudoxylaria* were grouped in clade TE, and species of the genera *Nemania* and *Rosellinia* were clustered in clade NR, in accordance with a previous study [36]. In HY clade, *X. schimicola* (FCATAS896), *X. theaceicola* (FCATAS903), and *X. wallichii* (FCATAS923), the three new species growing on fruits of *Schima* sp., were grouped together with high bootstrap support (85/1.0) with *X. liquidambaris*, associated with fruits of *Liquidambar formosana* in a subclade. In the PO clade, *X. rogersii* (FCATAS913, FCATAS915) and *X. vivantii* (HAST 519), two species growing on fruits of *Magnolia* sp., were grouped together with high bootstrap support values (97/1.0). In the *Xylaria* ITS tree (Figure 2), *X. schimicola* (FCATAS896, 898), *X. theaceicola* (FCATAS903, 904), and *X. wallichii* (FCATAS911, 923, 924) were grouped together with *X. liquidambaris* with weak support, whereas *X. rogersii* (FCATAS913, 914, 915) and *X. vivantii* (HAST 519) were grouped together with several other fructicolous *Xylaria* spp. with high support (90/-), with *X. rogersii* (FCATAS914) clustering at some distance from the other two specimens. Similarly, *Xylaria* species associated with fruits and seeds were distributed differently in three separate clades of the Xylariaceae ITS-RPB2-β-tubulin tree (Figure 1 and Figure S1) and the *Xylaria* ITS tree (Figure 2).

### 3.2. Taxonomy

**Figure 3:**
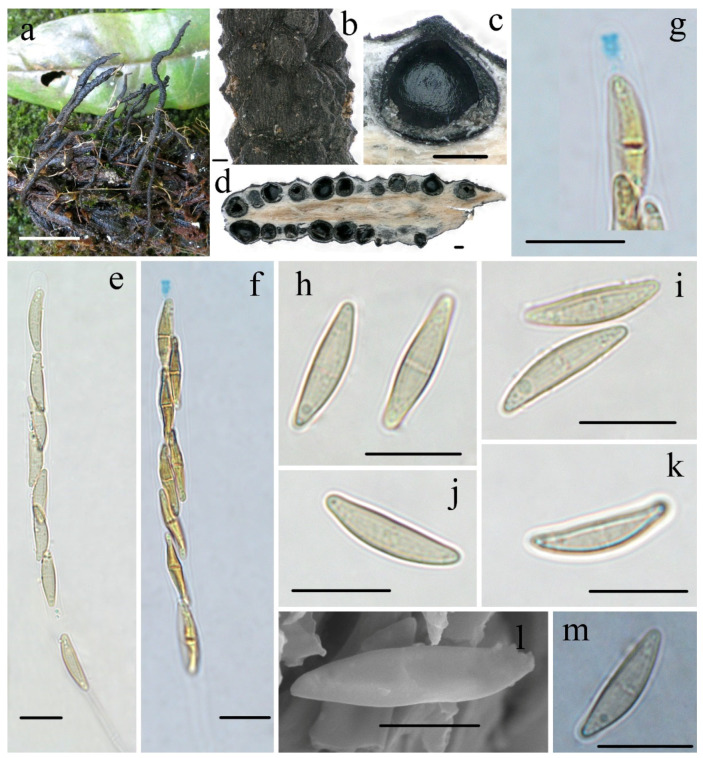
***Xylaria rogersii*** FCATAS915. (**a**) Stromata on fallen fruits. (**b**) Stromatal surface. (**c**,**d**) Section through stroma, showing perithecia. (**e**) Ascus in 1% SDS. (**f**) Ascus with ascus apical apparatus in Melzer’s reagent. (**g**) Ascus apical apparatus in Melzer’s reagent. (**h**) Ascospores with septa in water. (**i**) Ascospores in water. (**j**,**k**) Ascospore in 1% SDS. (**l**) Ascospore under SEM. (**m**) Ascospore in Indian ink. Scale bars: (**a**) = 2 cm; (**b**–**d**) = 200 µm; (**e**–**k**,**m**) = 10 µm; (**l**) = 5 µm.

***Xylaria rogersii*** Hai X. Ma & Yu Li, sp. nov., Figure 3.

MycoBank no: MB841144

**Etymology—***rogersii* (Lat.): Referring to American mycologist Prof. Jack D. Rogers, the leading world authority on the Xylariaceae who sadly passed away on 14 June 2021.

**Holotype—CHINA.** Yunnan Province, Honghe Hani Autonomous Prefectures, Pingbian County, Daweishan Nature Reserve, on fruits of *Magnolia* sp. (Magnoliaceae), 12 November 2019, Ma Haixia, Col. M31 (FCATAS915, GenBank accession: ITS = MZ648827, RPB2 = MZ707121, β-tubulin = MZ695800).

**Teleomorph—**Stromata upright or prostrate, solitary or sometimes clustered, unbranched or occasionally branched, with sterile apices, on long tomentose stipes, 5–12 cm total height; fertile parts 2–6 cm high × 1.5–3.0 mm broad, cylindrical, sometimes flattened, overlain with a dark-brown fine-striped outermost layer; stipes 14–60 mm high × 1.0–3.0 mm broad, terete, sometimes contorted, tomentose, with longitudinal wrinkles, arising from swollen base; surface black, roughened with half-exposed perithecial contours and striped outermost layer; interior light-yellow, woody. Perithecia subglobose, 400–600 µm in diam. Ostioles papillate. Asci eight-spored, arranged in uniseriate or partially biseriate manner, cylindrical, long stipitate, (100–)110–130(–140) µm total length, the spore-bearing parts (63–)70–80(–85) µm long × 5.0–6.0 µm broad, the stipes 30–55 µm long, with apical apparatus staining blue in Melzer’s reagent, urn-shaped to tubular, 2.2–2.6 µm high × 1.5–1.9 µm diam. Ascospores subhyaline to light-yellow, unicellular with a septum, inequilaterally naviform-ellipsoid, with tapered to narrowly rounded ends, sometimes slightly pinched, smooth, (13.0–)13.8–15.0(–15.6) × (3.3–)3.6–4.0(–4.4) µm (M = 14.4 × 3.7 µm, Q = 3.9, n = 90/3), without a discernable germ slit, lacking a sheath or appendages visible in Indian ink or 1% SDS.

**Additional specimen examined—CHINA.** Yunnan Province, Honghe Hani and Yi Autonomous Prefecture, Pingbian County, Daweishan Nature Reserve, on fruits of *Magnolia* sp. (Magnoliaceae), 12 November 2019, Ma Haixia, Col. M1 (FCATAS913, GenBank accession: ITS = MZ648825, RPB2 = MZ707119, β-tubulin = MZ695799), Col. M5 (FCATAS914, GenBank accession: ITS = MZ648826, RPB2 = MZ707120), Col. Z190, (FCATAS916).

**Notes—***Xylaria rogersii* was found on the fruits of *Magnolia* in Yunnan Province. It is characterized by long stromata with half-exposed perithecial contours and a dark-brown fine-striped outermost layer, with subhyaline to yellowish and unicellular ascospores that later form a septum. The specimens did not fit the descriptions of any known *Xylaria* species because of the ascospore septum. Rogers [6] described *Xylaria magnoliae* var. *magnoliae* from USA, which has a high specificity to fruits of *Magnolia* (Magnoliaceae). However, the Chinese collections are different from *X. magnoliae* var. *magnoliae*, which has subhyaline to yellowish ascospores lacking a discernable germ slit and long, tomentose stromatal surfaces [5,6]. Unfortunately, DNA sequences of the American material are not available in GenBank for phylogenetic analysis. However, the sequence comparison by Prof. Yu-Ming Ju (Institute of Plant and Microbial Biology, Academia Sinica, Taiwan, China) showed that there are 96.58%, 93.83%, and 95.35%, respectively, percent similarities in ITS, β-tubulin, and RPB2 between the Chinese material (FCATAS915) and *X. magnoliae* from the USA (J.D. Rogers RC8012, unpublished). Therefore, we described the Chinese material as a new species. The phylogenetic trees showed that *X. rogersii* and *X. vivantii* Y.M. Ju, J.D. Rogers, J. Fournier & H.M. Hsieh are sister species, forming a strongly supported branch, although *X. vivantii* is morphologically distinct due to its dichotomously branched stromata with a dark-brown tomentum, brown to dark-brown ascospores with an oblique germ slit surrounded by a hyaline sheath and bearing non-cellular appendages (Table 2).

**Figure 4:**
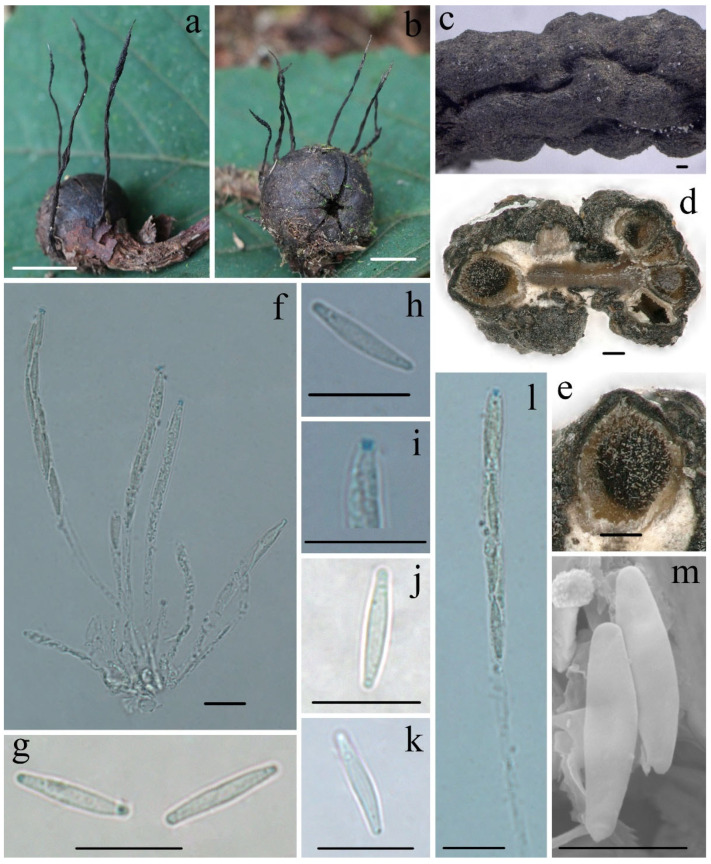
***Xylaria schimicola*** FCATAS896. (**a**,**b**) Stromata on fallen fruits. (**c**) Stromatal surface. (**d**,**e**) Section through stroma, showing perithecia. (**f**) Asci in Melzer’s reagent. (**g**) Ascospores in water. (**h**) Ascospore in Melzer’s reagent. (**i**) Ascus apical apparatus in Melzer’s reagent. (**j**) Ascospore in Indian ink. (**k**) Ascospore in 1% SDS. (**l**) Ascus with ascus apical apparatus in Melzer’s reagent. (**m**) Ascospores under SEM. Scale bars: (**a**,**b**) = 1 cm; (**c**–**e**) = 100 µm; (**f**–**l**) = 10 µm; (**m**) = 5 µm.

***Xylaria schimicola*** Hai X. Ma & Yu Li, sp. nov., Figure 4.

MycoBank no: MB840912

**Etymology—***Schimicola* (Lat.): Referring to the host genus *Schima*, which the fungus inhabits.

**Holotype—CHINA.** Yunnan Province, Jingdong County, Ailao Mountain Nature Reserve, on fruits of *Schima noronhae* Reinw. ex Bl. (Theaceae), 15 October 2013, Ma Haixia, Col. 17 (FCATAS896, GenBank accession: ITS = MZ648850, RPB2 = MZ707114, β-tubulin = MZ695787). 

**Teleomorph—**Stromata upright or prostrate, solitary or sometimes clustered, unbranched or occasionally branched from the stipes, (12–)20–50(–65) mm total height, with short to long thin stipes, tomentose when immature; fertile parts 4–26 mm high × 0.6–2.0 mm broad, narrowly fusiform to cylindrical with acute sterile apices up to 5 mm long, at times longitudinal furrowed, strongly nodulose with deep wrinkles isolating small groups of perithecia, more rarely furcate; stipes 7–50 mm high × 0.4–0.6 mm broad, smooth to downy, somewhat flattened, with longitudinal wrinkles, arising from a pannose, slightly enlarged base. Stroma surface smooth at young stage, white to cream-colored, black at mature stage, with inconspicuous to slight perithecial mounds, wrinkled, continuous, glabrous; interior whitish to buff (45) but dark-brown at center, solid, woody. Perithecia subglobose, 200–300 µm in diam. Ostioles faintly pronounced to papillate. Asci eight-spored, usually arranged in partially biseriate manner, cylindrical, long stipitate, (75–)85–95(–100) µm total length, the spore-bearing parts (41–)45–50(–55) µm long × (5–)5.5–6.5(–7.5) µm broad, the stipes 30–50 µm long, with apical apparatus staining blue in Melzer’s reagent, inverted hat-shaped to more or less rectangular, 0.7–1.3 µm high × 0.7–1.1 µm diam. Ascospores nearly hyaline to faintly light-yellow, unicellular, inequilaterally naviform-ellipsoid, with narrowly rounded ends, smooth, (9.5–)10.5–12.0(–13.0) × (1.6–)1.9–2.5(–3.0) µm (M = 11.2 × 2.2 µm, Q = 5.1, n = 60/2), lacking a discernable germ slit, no sheath or appendages visible in Indian ink or 1% SDS.

**Additionalspecimen examined—CHINA.** Sichuan Province, Mianning County, Lingshan Temple, on fruits of *Schima noronhae*, 12 July 2013, Ma Haixia Col. 259 (FCATAS898, GenBank accession: ITS = MZ648851).

**Notes—***Xylaria schimicola* was found on the fruits of *Schima noronhae* in the subtropics of Southwestern China, which did not fit the descriptions of any species known of genus *Xylaria* [5,6,7,12]. *Xylaria schimicola* is characterized by nearly hyaline to faintly light-yellow ascospores lacking a germ slit. The Chinese collections somewhat resemble *X. oxyacanthae* Tul. & Tul., *X. psidii* J.D. Rogers et Hemmes, and *X. palmicola* with winter-season stromatal morphology, but the ascospores are distinctly different [5]. In the phylogenetic trees, *Xylaria schimicola* formed a sister lineage with *X. wallichii* and *X. theaceicola*, both fruiting on pericarps of *Schima*.

**Figure 5:**
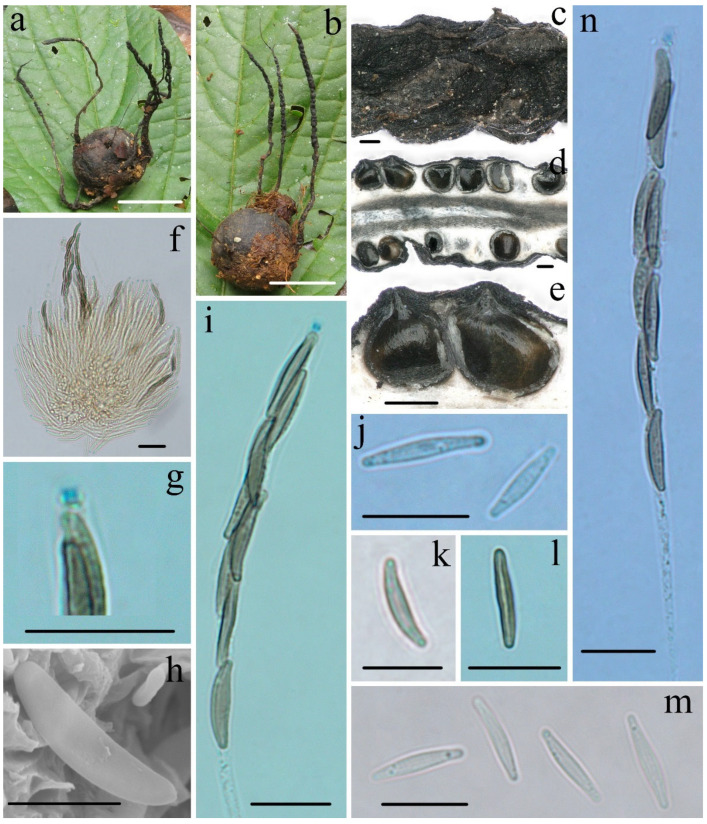
***Xylaria theaceicola*** FCATAS903. (**a**,**b**) Stromata on fallen fruits. (**c**) Stromatal surface. (**d**,**e**) Section through stroma, showing perithecia. (**f**) Asci in Melzer’s reagent. (**g**) Ascus apical apparatus in Melzer’s reagent. (**h**) Ascospore under SEM. (**i**,**n**) Asci and ascus apical apparatus in Melzer’s reagent. (**j**) Ascospores in water. (**k**) Ascospore in Indian ink. (**l**) Ascospore with germ slit in Melzer’s reagent. (**m**) Ascospores in 1% SDS. Scale bars: (**a**,**b**) = 1.5 cm; (**c**–**e**) = 200 µm; (**f**) = 20 µm; (**g**,**i**–**n**) = 10 µm; (**h**) = 5 µm.

***Xylaria theaceicola*** Hai X. Ma & Yu Li, sp. nov., Figure 5.

MycoBank no: MB840914

**Etymology—***theaceicola* (Lat.): Referring to the host family Theaceae, which the fungus inhabits.

**Holotype—CHINA.** Yunnan Province, Wenshan Zhuang and Miao Autonomous Prefecture, Wenshan County, Xiaoqiaogou Nature Reserve, on fruits of *Schima villosa* Hu (Theaceae), 16 November 2019, Ma Haixia, Col. M22 (FCATAS903, GenBank accession: ITS = MZ648848, RPB2 = MZ707115, β-tubulin = MZ695788). 

**Teleomorph—**Stromata upright or prostrate, solitary or sometimes clustered, unbranched or occasionally branched, with acute sterile apices, on a long, thin, ill-defined stipe, 2–8 cm total height; fertile parts 0.8–25 mm long × 0.5–1.5 mm broad, thin and cylindrical, usually crowded with perithecial contours slightly exposed, and occasionally with scattered perithecia, sometimes longitudinally furrowed, slightly nodulose with wrinkles isolating small groups of perithecia, more rarely furcate; stipes 1.2–6.5 cm high × 0.4–2 mm broad, smooth, with longitudinally wrinkled, arising from a pannose, slightly enlarged base; surface smooth at young stage, mature stromata black, with inconspicuous to slightly conspicuous perithecial mounds, overlain with a brown striped outermost layer; interior white, with a dark-brown to black circle, solid, woody. Perithecia subglobose, 300–450 µm in diam. Ostioles conical, papillate. Asci eight-spored, arranged in partially biseriate manner, cylindrical, long stipitate, (85–)92–105(–110) µm total length, the spore-bearing parts (52–)55–65(–70) µm long × (5.3–)5.5–6.5(–7.1) µm broad, the stipes are 25–53 µm long, with apical apparatus staining blue in Melzer’s reagent, inverted hat-shaped to tubular, 1.0–1.5 µm high × 0.8–1.2 µm diam. Ascospores faintly light-yellowish, nearly hyaline when immature, unicellular, ellipsoid, or navicular, arc-shaped, inequilateral, with broadly rounded ends, slightly pinched at the end, smooth, (10.1–)10.7–11.6(–12) × (2.0–)2.3–2.7(–2.9) µm (M = 11.1 × 2.5 µm, Q = 4.4, n = 60/2), with a straight germ slit along the spore length, lacking a slimy sheath visible in Indian ink or 1% SDS.

**Additional specimen examined—CHINA.** Yunnan Province, Wenshan Zhuang and Miao Autonomous Prefecture, Wenshan County, Xiaoqiaogou Nature Reserve, on fruits of *Schima villosa* (Theaceae), 16 November 2019, Ma Haixia, Col. Z193 (FCATAS904 GenBank accession: ITS = MZ648849).

**Notes—***Xylaria theaceicola* is characterized by long and usually unbranched stromata overlain with a brown, striped outermost layer, conical, papillate perithecial ostioles, faintly light-yellowish to nearly hyaline ascospores, with conspicuous straight germ slits, and growing on fruits of *S. villosa* (Theaceae). *Xylaria schimicola*, fruiting on pericarps of *S. noronhae*, is similar to *X. theaceicola* in that they share stromatal morphology, but differs on account of having ellipsoid ascospores lacking a discernable germination slit. The species also somewhat resembles *X. oxyacanthae, X. psidii*, and *X. palmicola* in stromatal morphology, but the ascospores of these species are distinctly different [5]. In the phylogenetic tree, *X. theaceicola* is a sister species to *X. schimicola*, but the relationship between the two fructicolous species of *Schima* is not strongly supported.

**Figure 6:**
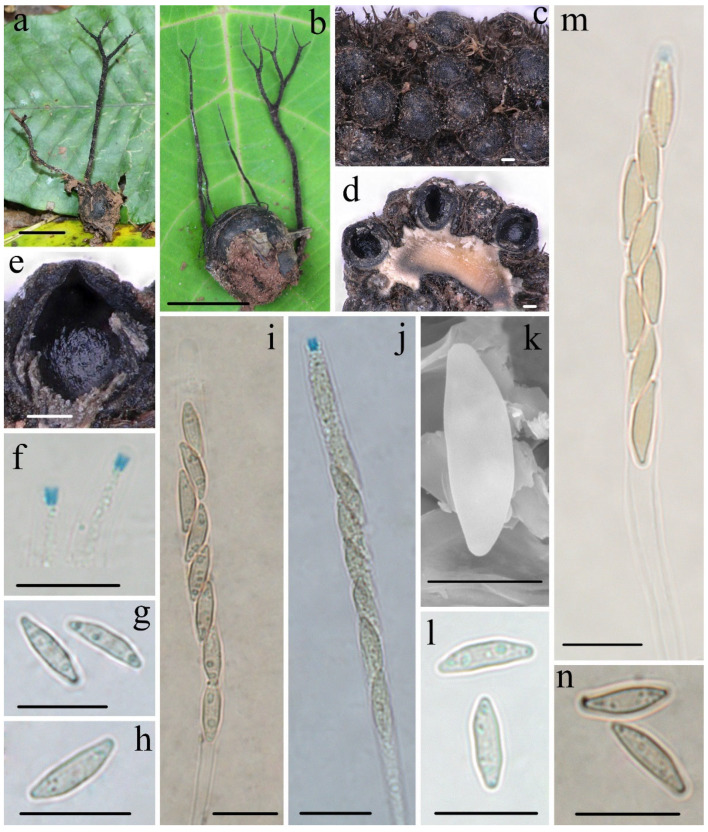
***Xylaria wallichii*** FCATAS911. (**a**,**b**) Stromata on fallen fruits. (**c**) Stromatal surface. (**d**,**e**) Section through stroma, showing perithecia. (**f**) Apical apparatus of asci in Melzer’s reagent. (**g**,**h**) Ascospores in water. (**i**) Ascus in Indian ink. (**j**,**m**) Asci with ascus apical apparatus in Melzer’s reagent. (**k**) Ascospore under SEM. (**l**) Ascospores in 1% SDS. (**n**) Ascospores in Indian ink. Scale bars: (**a**,**b**) = 1.5 cm; (**c**–**e**) = 100 µm; (**f**–**j**,**l–n**) = 10 µm; (**k**) = 5 µm.

***Xylaria wallichii*** Hai X. Ma & Yu Li, sp. nov., Figure 6.

MycoBank no: MB840915

**Etymology—***wallichii* (Lat.): Referring to the specific epithet of its host, which the fungus inhabits.

**Holotype—CHINA.** Yunnan Province, Jinghong City, Dadugang Town, on fruits of *Schima wallichii* (DC.) Choisy (Theaceae), 21 January 2015, Ma Haixia Col. 247 (FCATAS911, GenBank accession: ITS = ON222810, β-tubulin = MZ695797).

**Teleomorph—**Stromata upright or prostrate, solitary to sometimes densely clustered, often dichotomously branched several times, or infrequently unbranched, 1.5–10 cm total height, long stipitate; fertile parts 2–20 mm high × 1.0–2.0 mm broad, narrowly fusiform to cylindrical, often flattened, with acute sterile apices up to 5 mm long, strongly nodulose, mostly tomentose; stipes 13–80 mm high × 0.5–2.0 mm broad, terete to rarely flattened, often ill-defined, black-brown to black, conspicuously tomentose, arising from a slightly enlarged pannose base; surface roughened with perithecial mounds and tomentose except for stromatal apices, black; interior light-yellow to light-brown, black-brown in a circle, solid, woody. Perithecia subglobose, 300–400 µm in diam. Ostioles conical, papillate. Asci eight-spored, usually arranged in uniseriate manner, sometimes in partially biseriate manner, cylindrical, long stipitate, (75–)85–105(–115) µm total length, the spore-bearing parts (50–)55–63(–68) µm long × (4.1–)4.6–5.8(–6.2) µm broad, the stipes 25–50 µm long, with apical apparatus staining blue in Melzer’s reagent, inverted hat-shaped to more or less rectangular, 1.3–2.1 µm high × 1.1–1.7 µm diam. Ascospores nearly hyaline to light-yellow, unicellular, inequilaterally naviform-ellipsoid, with tapered to narrowly rounded ends, sometimes pinched on one end, smooth, (8.2–)8.8–10.2(–11.3) × (2.4–)2.6–3.0(–3.2) µm (M = 9.3 × 2.8 µm, Q = 3.3, n = 90/3), without a discernable germ slit, lacking sheath or appendages visible in Indian ink or 1% SDS.

**Additional specimen examined—CHINA.** Yunnan Province, Jinghong City, Dadugang Town, on fruits of *S. wallichii*, 21 January 2015, Ma Haixia, Col. 229 (FCATAS909), Col. 312 (FCATAS912); Dadugang Town, Guanping Village, on fruits of *S. wallichii*, 21 January 2015, Ma Haixia, Col. 234 (FCATAS910); Yunnan Province, Pu’er City, Taiyanghe National Forest Park, on fruits of *S. wallichii*, 18 October 2013, Ma Haixia, Col. 18 (FCATAS923, GenBank accession: ITS = MZ648861, RPB2 = MZ707118, β-tubulin = MZ695793), Col. 30 (FCATAS924, GenBank accession: ITS = MZ648862, β-tubulin = MZ695794).

**Notes—**So far, *Xylaria wallichii* has only been found on fruits of *S. wallichii* (Theaceae) from the tropics and the transitional zone from the subtropics to tropics. This species is characterized by almost hyaline ascospores that lack a germ slit, a sheath, or appendages, and with stromata often dichotomously branched several times covered by conspicuously tomentose and perithecial mounds. The three species of the present study, *X. wallichii*, *X. schimicola*, and *X. theaceicola*, found on fruits of the genus *Schima*, have similar hyaline or nearly hyaline ascospores and form a common clade in the phylogenetic trees. However, they are clearly distinguishable based on the branching of stromata, presence or absence of germ slits, and the shape and size of ascospores. *Xylaria magnoliae* var. *magnoliae* also has pale-colored ascospores without a discernable germ slit and sheath, but differs in that is has larger ascospores (12.5–)13.5–15(–16) × (2.5–)3–3.5(–4) µm (M = 14.1 × 3.2 µm), unbranched or occasionally branched stromata, and grows on pericarps of *Magnolia* species (Magnoliaceae) [5]. Three other taxa, *X. apeibae* Mont., *X. xanthinovelutina*, and *X. reevesiae* Y.M. Ju, J.D. Rogers & H.M. Hsieh are somewhat similar to *X. wallichii* in stromatal morphology, but differ in their ascospores [5]. *Xylaria apeibae* has light-brown and larger ascospores (9.5–)10–12(–13) × (3–)3.5–4(–4.5) µm (M = 11.0 × 3.7 µm), with a straight germ slit and grows on fruits of *Apeiba* species (Tiliaceae) [13]. *Xylaria xanthinovelutina* has brown and slightly larger ascospores (9–)9.5–11(–12) × (3.5–)4–4.5(–5) µm (M = 10.3 × 4.0 µm), with a straight germ slit, a hyaline sheath, and non-cellular appendages, and grows on leguminous pods. *Xylaria reevesiae* has brown and slightly larger ascospores (8.5–)9–10.5(–11) × (4–)4.5–5.5(–6) µm (M = 9.7 × 5.0 µm), with a straight germ slit, and grows on fruits of *Reevesia formosana* (Sterculiaceae) [5]. Phylogenetically, *X. wallichii* is distinct from all the *Xylaria* species mentioned.

## 4. Discussion and Conclusions

Previous investigations have discovered several new species in Southwest China [43,44], and the current study confirmed the unexplored species diversity of the area. Here, four pale-spored *Xylaria* species from Southwest China were introduced as new taxa based on morphological characteristics, host association, and phylogenetic analyses. Combined ITS, RPB2, and β-tubulin sequence data of a representative sample of the entire genus showed that the four new species are distributed in two distinct lineages of the phylogenetic tree. Considering all known species associated with fallen fruits and seeds, fructicolous taxa formed clusters in three different clades. This suggests that the fructicolous life style of *Xylaria* species has evolved independently several times within the genus *Xylaria*. Moreover, the texture of the fruits or seeds may have promoted or influenced speciation, as reflected by the phylogenetic relationships of *Xylaria* species associated with fallen fruits and seeds. To further test this hypothesis, it is crucial to carry out additional studies and confirm the phylogenetic position of all *Xylaria* species associated with fallen fruits and seeds. 

Many xylariacean endophytes are a source of bioactive secondary metabolites with antibacterial, antioxidative, anti-carcinogenic, and other properties [45,46]. Unfortunately, we could not obtain cultures from these isolates, and thus, they were not accessible for phylogenetic studies. Future research should include additional specimens of *Xylaria* from different hosts and substrates using an integrative approach including morphological, chemotaxonomic, and phylogenetic data.

## Figures and Tables

**Table 2:** A dichotomous key to worldwide species of *Xylaria* associated with fruits and seeds.

1. Ascospores pale or subhyaline	2
1. Ascospores brown to dark brown	7
2. Stromata tomentose on the fertile part	3
2. Stromata glabrous on the fertile part	5
3. Stromata with half- to fully exposed perithecial mounds, frequently dichotomously branched; ascospores (8.5–)9–10.5(–11) × (4–)4.5–5.5(–6) µm	***X. wallichii***
3. Stromata with inconspicuous perithecial mounds, unbranched in most cases	4
4. Ascospores (12.5–)13.5–15(–16) × (2.5–)3–3.5(–4) µm	*X. magnoliae* var. *magnoliae* *
4. Ascospores (7.5–)8–9(–10) × (2.5–)3–3.5(–4) µm	*X. magnoliae* var. *microspore* *
5. Ascospores with a conspicuous straight germ slit	***X. theaceicola***
5. Ascospores without a discernible germ slit	6
6. Stromata associated with fruits of *Magnolia* (Magnoliaceae); ascospores (13.0–)13.8–15.0(–15.6) × (3.3–)3.6–4.0(–4.4) µm	***X. rogersii***
6. Stromata associated with fruits of *Schima noronhae* (Theaceae); ascospores (9.5–)10.5–12.0(–13.0) × (1.6–)1.9–2.5(–3.0) µm	***X. schimicola***
7. Stromata tomentose on the fertile part	8
7. Stromata glabrous on the fertile part	15
8. Ascospores lacking a hyaline sheath and appendages	9
8. Ascospores surrounded by a hyaline sheath and bearing non-cellular appendages at ends	10
9. Stromata associated with fruits of *Apeiba* (Tiliaceae); ascospores (9.5–)10–12(–13) × (3–) 3.5–4(–4.5) µm	*X. apeibae* *
9. Stromata associated with fruits of *Bauhinia cumingniana* (Fabaceae); ascospores (8–)8.5–9.5(–10) × 3–3.5(–4) µm	*X. luzonensis* *
10. Ascospores with an oblique germ slit	11
10. Ascospores with a straight germ slit	12
11. Stromata associated with fruits of *Magnolia* (Magnoliaceae); ascospores brown to dark-brown, ellipsoidal-inequilateral, (14.5–)15–16.5(–17.5) × 4.5–5.5(–6) µm	*X. vivantii* *
11. Stromata associated with fruits of *Elizabetha* pod; ascospores brown, fusoid-inequilateral, (14–)14.5–16(–17) × (3.5–)4–4.5(–5) µm	*X. rossmanae* *
12. Ascospores (11–)11.5–13.5(–14.5) × (4–)4.5–5(–5.5) µm	*X. patrisiae* *
12. Ascospores mostly smaller than 11.5 µm	13
13. Stromata associated with capsules of *Cullenia excelsa* (Malvaceae); ascospores (7.5–)8–9(–9.5) × (3.5–)4–4.5(–5) µm	*X. culleniae* *
13. Stromata associated with other substrates	14
14. Ascospores (9–)9.5–11(–12) × (3.5–) 4–4.5(–5) µm	*X. xanthinovelutina* *
14. Ascospores 7.5–10 × 3.4–4.8 µm	*X. fabaceicola* **
15. Stromata capitate, subglobose, or obconical	16
15. Stromata cylindrical to filiform	18
16. Stromata associated with decaying leaves of *Clusia* (Clusiaceae); ascospores (11.6–)12.8–16.7(–18) × 8–15 µm	*X. clusiae* *
16. Stromata associated with fruits of other substrates	17
17. Stromata associated with fruits of *Gluazuma*; ascospores (15–)15.5–16.5(–17) × (5.5–)6–7(–7.5) µm	*X. guazumae* *
17. Stromata associated with different substrates; ascospores (14.5–)15.5–18(–19) × (5–)5.5–6.5(–7) µm	*X. heloidea* *
18. Ascospores surrounded by a hyaline sheath and bearing non-cellular appendages at ends	*X. aethiopica* ^#^
18. Ascospores lacking a hyaline sheath	19
19. Stromata filiform	20
19. Stromata cylindrical	22
20. Stromata growing on leaves and fruit remains of *Quercus polymorpha* (Fagaceae); ascospores 12–14.5(–16) × 4–4.5(–5) µm	*X. duranii* *
20. Stromata associated with fruits or seeds	21
21. Stromata associated with dead angiospermous seeds; ascospores (11.5–)13–15(–15.5) × (4.5–)5–5.5(–6) µm	*X. himalayensis* *
21. Stromata associated with *Sloanea* capsules; ascospores (9.5–)10–11.5(–12.5) × (3.5–)4–4.5(–5) µm	*X. warburgii* *
22. Stromata associated with pericarps or pods	23
22. Stromata associated with fruits or seeds	24
23. Stromata associated with pericarps *Fagus sylvatica* (Fagaceae); ascospores (9.5–)11–12(–13) × (4–)4.5–5(–5.5) µm	*X. carpophila* *
23. Stromata associated with decaying seed pods of Fabaceae; ascospores 9–11.2 × 3–4.3 µm	*X. fabacearum* **
24. Stromata lacking a striped outer layer	25
24. Stromata overlain with a striped outer layer	27
25. Stromata with perithecial mounds fully exposed; ascospores (9.5–)10.5–11.5(–12.5) × (5–)5.5–6.5(–7) µm	*X. jaliscoensis* *
25. Stromata with perithecial mounds inconspicuous to protuberant	26
26. Stromata associated with seeds of *Chlorocardium rodiei* (Lauraceae); ascospores (8–)8.5–9.5(–10.5) × 3.5–4(–4.5) µm	*X. karyophthora* ^##^
26. Stromata associated with fallen fruits of *Beilschmiedia percoriacea* (Lauraceae); ascospores (11–)12–14 × 4–5(–6) µm	*X. beilschmiediae* ^###^
27. Ascospores with a spiral germ slit, (13–)13.5–15(–16) × (4.5–)5–5.5(–6) µm; on fruits of *Liquidambar* (Altingiaceae)	*X. liquidambaris* *
27. Ascospores with a straight germ slit	28
28. Stromata associated with fallen fruits	29
28. Stromata associated with seeds	30
29. Stromata associated with fallen fruits of *Euphorbia* (Euphorbiaceae); ascospores (8–)8.5–9.5(–10) × (3.5–)4–5(–5.5) µm	*X. euphorbiicola* *
29. Stromata associated with fallen fruits of *Reevesia formosana* (Sterculiaceae); ascospores (8.5–)9–10.5(–11) × (4–)4.5–5.5(–6) µm	*X. reevesiae* *
30. Stromata associated with palm seeds	31
30. Stromata associated with seeds of dicots	32
31. Stromata associated with seeds of *Euterpe globosa*; ascospores (13.5–)14.5–16.5(–18.5) × (6–)6.5–7.5(–8.5) µm	*X. palmicola* *
31. Stromata associated with palm seeds; ascospores (9.5–)10.5–12(–13.5) × (4–)4.5–5.5(–6) µm	*X. rhizocola* *
32. Stromata associated with seeds of *Psidium guajava* (Myrtaceae); ascospores (8.5–)9–10.5(–12) × (4–)4.5–5(–5.5) µm	*X. psidii* *
32. Stromata associated with seeds of various plants; ascospores (9.5–)10–11.5(–12) × (4–)4.5–5.5(–6) µm	*X. oxyacanthae* *

* See Ju et al. [5], ** see Perera et al. [8], ^#^ see Fournier et al. [11], ^##^ see Dillon et al. [12], ^###^ see Huang et al. [24].

## Data Availability

All newly generated sequences were deposited in GenBank (https://www.ncbi.nlm.nih.gov/genbank/, accessed on 7 March 2022; Table 1). Data for all new taxa were deposited in MycoBank (https://www.mycobank.org/, accessed on 5 March 2022; MycoBank identifiers follow new taxa).

## Supplementary Materials

The following are available online at https://www.mdpi.com/article/10.3390/biology11060885/s1, Figure S1: Phylogenetic tree of *Xylaria* and related genera based on the multigene alignment of ITS-RPB2-β-tubulin in the Bayesian tree. Bayesian posterior probabilities (PP) ≥ 0.95 are labeled above or below the respective branches. Species in bold were sequenced in this study.
