# Supplementary figures and images for: Four New Pale-Spored Species of Xylaria (Xylariaceae, Xylariales) with a Key to Worldwide Species on Fallen Fruits and Seeds
Source: Biology (Basel). 2022 Jun 8;11(6):885. doi: 10.3390/biology11060885 (PMC9220042; doi:10.3390/biology11060885)

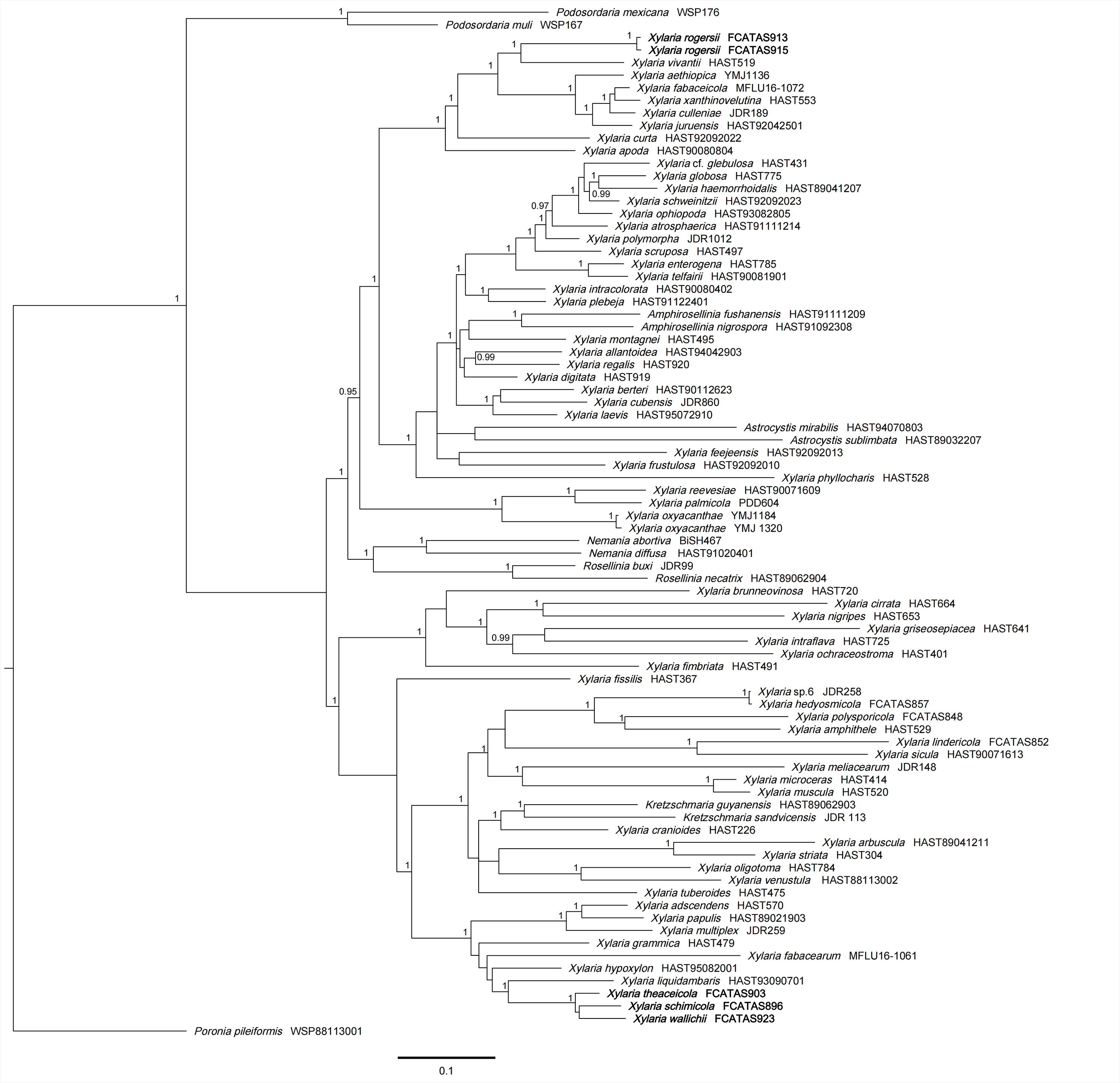

Supplement: Supplementary file 1 [file biology-11-00885-s001.zip › Figure S1.tif]
